# Deep neck space infections of odontogenic origin are costly and preventable

**DOI:** 10.2340/aos.v83.41382

**Published:** 2024-09-13

**Authors:** Christina Hellgren, Johan Hellgren, Behnosh Öhrnell Malekzadeh

**Affiliations:** a Clinic of General Dentistry Järntorget, Public Dental Service, Region Västra Götaland, Gothenburg, Sweden; b Department of Oral and Maxillofacial Radiology, Institute of Odontology, Sahlgrenska Academy, University of Gothenburg, Gothenburg, Sweden; c Department of Otorhinolaryngology, Head & Neck Surgery, Institute of Clinical Sciences, Sahlgrenska University Hospital, University of Gothenburg, Gothenburg, Sweden; d Department of Otorhinolaryngology, Head & Neck Surgery, Sahlgrenska University Hospital, Region Västra Götaland, Gothenburg, Sweden; e Clinic of Oral and Maxillofacial Surgery, Public Dental Service, Region Västra Götaland, Borås, Sweden; f Department of Oral and Maxillofacial Surgery, Institute of Odontology, Sahlgrenska Academy, University of Gothenburg, Gothenburg, Sweden

**Keywords:** Deep neck space infection, odontogenic, cost of illness, direct costs, indirect costs

## Abstract

**Objective:**

Deep neck space infections (DNSI), caused by the spread of an odontogenic infection to the floor of the mouth and neck, are potentially life-threatening but preventable. We explored the total cost of illness (COI) for patients with DNSI of odontogenic origin.

**Material and methods:**

Cross-sectional, register-based, multi-centre study of the health economics of DNSI treatment. Included were patients aged > 18 years who were treated in hospital for DNSI of odontogenic origin. Subjects were identified from the regional healthcare database VEGA based on the International Classification of Diseases (ICD) codes and surgical procedure codes. The cost per patient (CPP) values for the hospital care, prescription medications and sick leave were extracted.

**Results:**

In total, 148 patients were included. The average length of the hospital stay was 6 days. Total COI was estimated as 15,400 EUR per patient and 2,280,000 EUR in total. Direct costs accounted for 93% of the COI, and indirect costs were 7%.

**Conclusion:**

The total COI for patients with DNSI of odontogenic origin was six-fold higher than the average COI for patients in otorhinolaryngology (ORL) care. Preventing DNSI will entail substantial cost savings for the specialised healthcare units and will have a significant impact on the patients.

## Introduction

A deep neck space infection (DNSI) is a severe complication of a local infection in the mouth, throat or the teeth, including infection following a dental procedure, which spreads into the floor of the mouth and into the deep compartments of the neck [1,2]. If left untreated, DNSI is fatal in more than 60% of patients [3]. For patients with DNSI who do receive treatment, the mortality rate is 5% [4], which increases to 20–30% if intensive care is needed [5] or if tracheotomy is required [6]. Severe complications of DNSI include airway obstruction, sepsis, cervicofacial necrotising fasciitis, internal jugular vein thrombosis and mediastinitis [7].

It has been estimated that DNSIs of odontogenic origin affect 47.7% of all patients with DNSI, thus representing the majority of all patients with DNSI [1]. The odontogenic origin may involve apical periodontitis, periodontitis, pericoronitis and complications after dental procedures, such as tooth extraction and other odontogenic interventions. Other sites of origin of DNSI are the submandibular gland and the tonsils.

Patients with DNSI are resource-demanding for healthcare providers. Knowledge of the costs related to DNSI is important in order to plan and allocate healthcare resources. In Sweden, as in many other countries, patients with DNSI are admitted to an otorhinolaryngology (ORL) clinic, and most of these patients are treated with a combination of antibiotics and surgery. In the case of DNSI of odontogenic origin, a team of ORL surgeons and oral surgeons treats the patients. The complex anatomy of the floor of the mouth and the neck and its different muscular spaces and compartments and the proximity with the airway and several larger blood vessels and nerves, makes DNSI a clinical challenge. It involves the need to acquire an accurate radiological and clinical picture of the extent and spread of the infection and to choose the appropriate antibiotics and surgical approach. A mean hospital stay of 6.6 days has been reported [8], and having patients in hospital care has been shown to be particularly resource demanding. In 2017, the total costs associated with all inpatient care in Sweden were estimated at 9.5 billon EUR (102 billion SEK [Swedish kronor]), corresponding to approximately 20% of total healthcare expenditures in Sweden [9]. It is, therefore, reasonable to assume that the treatment of DSNI, which requires patients to spend up to 1 week in hospital, is associated with high costs. However, this aspect has not been previously studied in a comprehensive way. In a study from Canada involving a small patient sample (*N* = 9), the direct costs per patient with DNSI requiring surgical drainage amounted to 21,639 USD in 2013 [10]. In a study conducted in Australia that included 462 patients with DNSI of odontogenic origin, the mean cost per patient (CPP) was 12,228 AUD in 2020 [11].

The aim of the present study was to estimate the total cost of illness (COI) based on data acquired from several unique databases related to individual healthcare utilisation in Sweden. The COI analyses includes both *direct costs* generated by the hospital care and the medicines, and *indirect costs* linked to the patients being unable to perform work or studies during their illness, as well as the distribution of these costs.

## Methods

This is a cross-sectional, register-based study of the total COI for all adult patients with DNSI of odontogenic origin who were treated in a hospital in the Västra Götaland region (VGR) in 2019. The VGR is the second-largest region in Sweden, with a population of 1.71 million (year 2019). Five major hospitals provide specialised emergency healthcare for patients with DNSI. Since this is a register-based study, no informed consent was needed, in accordance with the approval granted by the Swedish Ethical Review Authority (Dnr. 2021-04621).

### Inclusion criteria

All patients aged > 18 years registered in the VEGA database (see below) for in-hospital care of a DNSI of odontogenic origin in the VGR in 2019 were eligible. They also had to meet one or both of the following criteria: (1) hospitalisation at an ORL clinic in the VGR; or (2) collected at least one course of prescribed antibiotics aimed at treatment of a DNSI of odontogenic origin within 3 days before hospitalisation or 1 day after discharge (based on data from the Digitalis database).

Deep neck space infection of odontogenic origin was identified in the VEGA database with the International Statistical Classification of Diseases and Related Health Problems (ICD)-10 codes or the medical/surgical procedure codes, the so-called KVÅ *(klassifikation av åtgärd-KVÅ) codes* used in the VGR. The ICD-10 codes used for inclusion were; K.04.0, pulpitis; K04.4, acute apical periodontitis of pulpal origin; K04.5, chronic apical periodontitis; K04.6, periapical abscess with sinus; K04.7, periapical abscess without sinus; K10.2, inflammatory conditions of jaws; and K12.2, cellulitis and abscess of mouth.

The KVÅ codes used for inclusion were: ECA10, incision and drainage through gingiva; EDA00, incision lower jaw; EEA00, incision upper jaw; EHA00 incision palate, EJA00, incision tongue or floor of the mouth; and QAA10, incision head-neck region.

Patients with ICD-10 codes related to infections originating from the submandibular gland or the tonsils were not included.

Prescribed antibiotics were identified in the Digitalis database with the Anatomical Therapeutic Chemical (ATC) codes: J01CF05, J01FF01, J01CA08, J01FF01, J01EE01, J01MA02, J01CR02, J02AC01, J01XE01, J01DB05, J01XA02, J05AB01, J01MA14, J01XC01, J05AB11, J02AC04, J01AA04, J01EA01, J01FA01, and J05AH02.

### Databases

VEGA is a regional, administrative healthcare database that contains data on all healthcare contacts (primary, specialised, outpatient and inpatient) registered for the population of the VGR. The database also includes information on the residential address, age and sex of each subject, and an estimated individual CPP related to their healthcare use.

Digitalis is a regional database of all drugs collected at a pharmacy, as prescribed for the population in the VGR (within or outside the VGR). Medications are categorised according to the ATC classification system.

*Försäkringskassan* (The Swedish Social Insurance Agency) is a tax-financed, national public insurance authority that provides welfare during sick leave. The first 14 days of sick leave are paid directly by the employer and are not registered in this system.

### COI

The COI is a validated tool for describing the economic consequences of diseases (SBU – Swedish Agency for Health Technology Assessment and Assessment of Social Services, 2017). Both *direct* and *indirect* costs are included in the COI.

### Direct costs and CPP

Direct costs are the costs for hospitalisation, surgery and medicines used in hospital care, as well as the costs related to outpatient visits. The direct costs were extracted from the VEGA database as CPP. Cost per patient is a validated method for calculating healthcare costs per contact and patient in Sweden [12]. It includes information on the type of care provided and the resources used at each care contact in the outpatient and inpatient settings, including expenses related to premises, medical equipment, health personnel, materials, sampling, examinations, drugs administered while admitted to the hospital, administration, among others. Excluded from the CPP are costs related to ambulance services, healthcare journeys (between home and the healthcare provider), prescription drugs and the specialised dental care, including oral surgery and oral radiology (computed tomography [CT] scan performed by neuroradiology is included in the CPP, but not additional oral radiology when needed).

The costs for collected medications that were prescribed within 3 days before admittance to the hospital and 1 day after discharge from the hospital were identified in the Digitalis database.

Any visit to the ORL department for outpatient care within 14 days after leaving the hospital in connection with a DNSI diagnosis was classified as care related to the DNSI diagnosis and was included in the direct costs using CPP.

The cost for oral surgery is not included in the CPP. This cost was calculated on the basis of the average total estimated time spent on a typical patient with DNSI of odontogenic origin not requiring intensive care, up to a mean of 10 hours (excluding stand time). This included the costs per hour for a dental specialist and nurse, based on the hourly wage costs in the Swedish public dental care system (*Folktandvården, VGR*) in 2019.

### Indirect costs

The indirect costs were calculated using the human capital approach [13]*.* According to this approach, the indirect costs from sick leave were calculated using the average future earnings of the patients while on sick leave. For the majority of the patients who had sick leave shorter than 14 days, these costs were based on age- and sex-adjusted mean wages given by Statistics Sweden plus the additional social security contributions made by the employers. The days spent in hospital care were regarded as sick leave and calculated accordingly. For the patients who had sick leave longer than 14 days, the specific individual costs were provided by the Swedish Social Insurance Agency. Additional costs for the first 14 days were then added for these patients, as they are required in order to obtain reimbursement from the Swedish Social Insurance Agency.

The main results from this study are presented, using the annual average exchange rate from the Swedish Central Bank for year 2019 (1 EUR = 10.5892 SEK).

### Statistical analysis

Descriptive tables are provided for all the variables. Continuous variables are presented as means or medians, with standard deviations and, where appropriate, the sum. Categorical variables are presented as frequencies [number of cases (N)] and percentages (%), or only as percentages of the total number of patients observed in each category. A COI analysis has been applied using the Stata ver. 17.0 software (Stata Corp LLC, 2017). As some individuals had missing data on the CPP in primary and specialised outpatient care, it was necessary to impute the costs.

## Results

**Figure 1:**
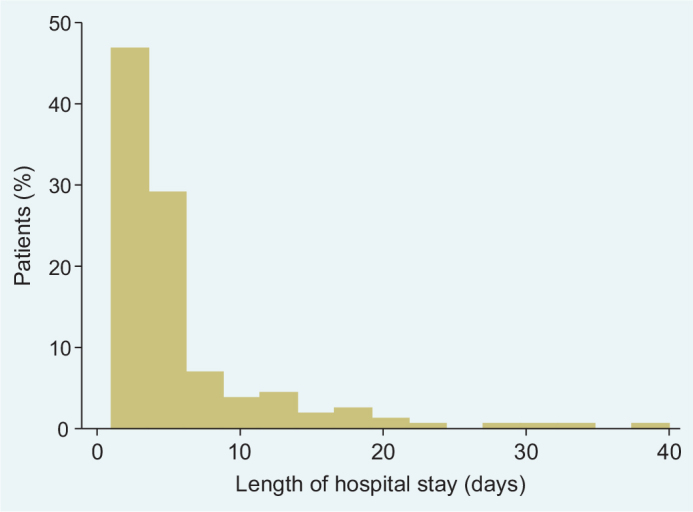
Length of stay in hospital (days) for the patients with deep neck space infections (DNSI) in Västra Götaland region (VGR) 2019 *n* = 148, % of patients on the y-axis.

In total, 148 individuals with DNSI of odontogenic origin (mean age, 51.1 years; 53.4% women) were included in the study. More than 80% of the patients were hospitalised at an ORL clinic in the VGR. In all, 139 (94%) had one hospitalisation, 8 had two hospitalisations, and 1 patient had three hospitalisations for DNSI in 2019. The average length of the in-hospital care for DNSI was 6 days (range, 1 to 40 days) (Figure 1). The mean total number of collected prescriptions for antibiotics was 3. Overall, 12 individuals had DNSI-related sick leave benefits exceeding 14 days and 1 individual had two separate sick leave periods, resulting in a total of 13 observations. The number of sick leave days spent in hospital ranged from 17 days to 365 days, with a mean of 59 days (median, 21 days).

### Costs

**Figure 2:**
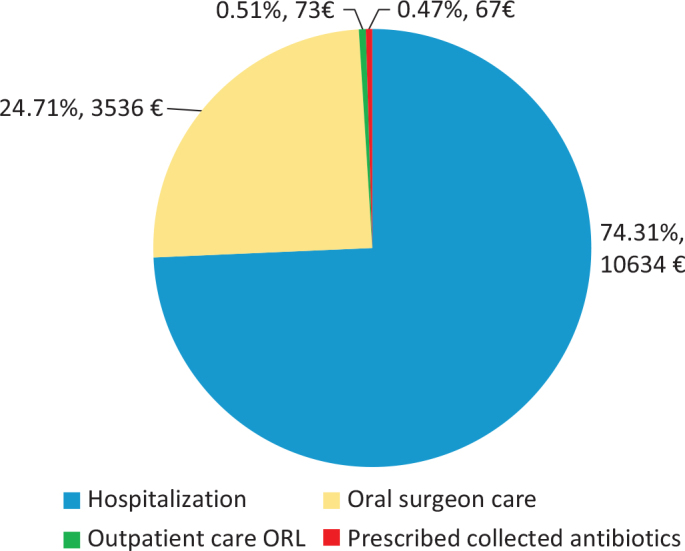
Mean cost per patient (CPP) in Swedish kronor (SEK) in 2019 value per patient with DNSI of odontogenic origin hospitalised in Västra Götaland region (VGR). Costs are stratified by cost for hospitalisation, cost for oral surgeon care., out-patient follow up visits and collected prescribed medicines. Cost in SEK and % of total mean CPP. ORL: otorhinolaryngology.

The total COI was estimated at 15,433 EUR (163,411 SEK) per patient with DNSI of odontogenic origin, and 2,284,014 EUR (24,000,000 SEK) in total. The mean direct and indirect costs as well as the total COI are shown in Table 1 and Figure 2. The total productivity losses were 166,010 EUR (1,760,000 SEK) for the 148 patients with DNSI, with a mean cost of 1,122 EUR (11,877 SEK) per patient. The total production loss following sick leave for the first 14 days was estimated as 39,427 EUR (417,500 SEK).

**Table 1:** COI per patient with DNSI of odontogenic origin (*n* = 148) treated in hospital in VGR 2019, including direct, based on CPP, and indirect costs.

COI	Cost component	Mean CPP (SEK)	Total cost for all patients (SEK)	Mean CPP (EUR)	Total cost all patients (EUR)	% of total COI
Direct costs	Prescription drug use (antibiotics)^1^	715	105,755	67	9,987	0.4
	Follow-up outpatient visit (ORL)	778	115,163	73	10,876	0.5
	In hospital care	112,601	16,665,008	10,634	1,573,843	68.9
	Oral surgeon care	37,440	5,541,060	3,536	523,298	22.9
	Total direct costs	151,534	22,426,986	14,311	2,118,005	92.7
Indirect costs	Production loss, > 14 days of sick leave	9,056	1,340,313	855	126,579	5.5
	Production loss, 14 first days of sick leave	2,821	417,522	266	39,431	1.7
	Total indirect costs	11,877	1,757,835	1,122	166,010	7.3
Cost-of-illness	Total COI, 2019	163,411	24,184,821	15,433	2,284,014	100.0

The costs are presented in SEK and euros (€).

Note: Price Year 2019, 1 EUR = 10.589 SEK.

1 ATC codes: J01CF05, J01FF01, J01CA08, J01FF01, J01EE01, J01MA02, J01CR02, J02AC01, J01XE01, J01DB05, J01XA02, J05AB01, J01MA14, J01XC01, J05AB11, J02AC04, J01AA04, J01EA01, J01FA01, J05AH02.

CPP: cost per patient; SEK: Swedish kronor; COI: cost of illness; ORL: otorhinolaryngology; VGR: Västra Götaland region; DNSI: deep neck space infections; ATC: Anatomical Therapeutic Chemical.

**Figure 3:**
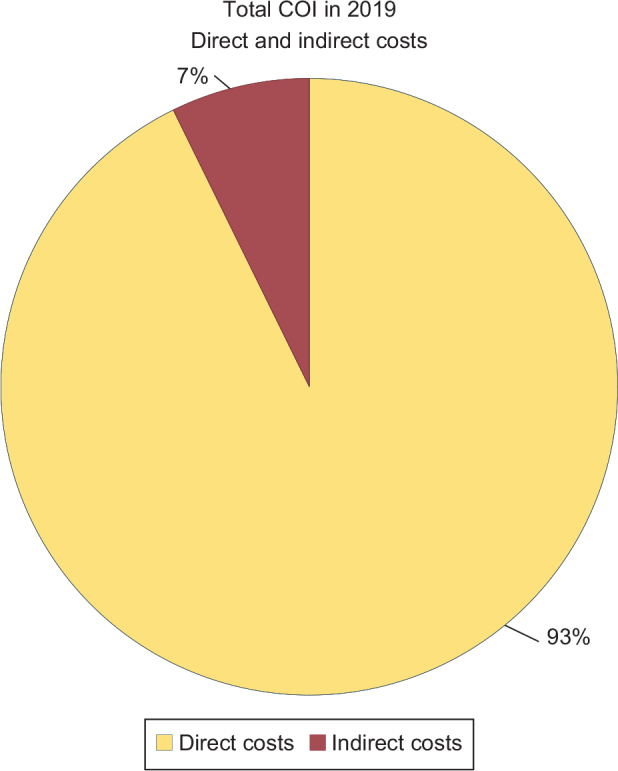
The distribution of total cost of illness (COI) on direct health care costs and indirect costs for lost production in 148 hospitalised patients with deep neck space infections (DNSI) of odontogenic origin in Västra Götaland region (VGR), Sweden in 2019.

Indirect costs accounted for 7% and direct healthcare costs for 93% of the total COI (Figure 3). The largest component of the total COI was inpatient care, accounting for 69% of the total COI.

When adjusted to the year 2023 Swedish price level (Statistics Sweden, 2024), the mean COI per patient was 17,187 EUR (197,324 SEK).

## Discussion

Deep neck space infections of odontogenic origin, which is a potentially preventable and serious disease, is in this study associated with a mean total COI of 15,433 EUR (163,411 SEK) per patient. This is six-fold higher than the average COI for patients in the VGR. More than 90% of the COI was *direct costs* borne by the healthcare system itself and, thus, affecting other in-hospital healthcare. Early detection of patients at risk of developing DNSI could render substantial cost savings for the specialised healthcare units and significantly reduce the level of suffering for the patients.

The total COI calculated in the present study is comparable to the COI values reported by Han et al. and Biron et al. [10,11], and confirm that DNSI is costly especially if being of odontogenic origin. Since 93% of the total COI was direct costs, this means that most of the costs are carried by the healthcare system directly. This affects the ability to deliver other healthcare provided by involved in-hospital resources including oral surgery and ORL. Patients with DNSI are typically admitted as emergency cases. With an average in-hospital stay of 6 days of unplanned highly specialised care, these patients are a challenge both from the medical, odontological and financial points of view. The average in-hospital stay for all ORL patients in the VGR is two nights. The need for emergency surgical procedures and other advanced care for cases of DNSI can result in the cancellation or delay of planned surgery, as the hospital and oral surgeon’s resources are limited. Therefore, it is important for healthcare planners and decision-makers to be aware of the impact of this disease. While patients with DNSI of odontogenic origin are initially in outpatient care, when they progress from outpatient care to in-hospital care, the COI rises. According to statistics from the VGR, the average healthcare cost (including direct and indirect costs) per resident was 2,421 EUR (25,634 SEK) in 2019. This cost was calculated based on the average healthcare expenses for the whole population in the VGR. The mean COI per patient with DNSI in 2019 was almost six-fold higher. In Sweden, patients with DNSI are typically admitted to an ORL clinic. The five ORL clinics that treat patients with DNSI in the VGR have 6–16 beds available per night to cover the needs of all acute and elective ORL patients who require hospital care. The mean duration of hospital stays for the 148 patients in this study corresponds to allocating all the available ORL in-hospital beds in the VGR to patients with DNSI for 4 days in a single year.

A major strength of the present study is the available register data from several regional and national databases. The unique VEGA register contains all patient contacts with healthcare within the VGR, as well as the linked individual CPP values. The registration of these data (the ICD-10 KVÅ codes) is mandatory, therefore missing data are unlikely. We defined DNSI according to the codes related to odontogenic infections used in the VGR when treating DNSI of odontogenic infection in hospitalised patients. The most common ICD-10 code used for DNSI of odontogenic origin in the VGR is K12.2, cellulitis and abscess of mouth (*N* = 60), as a single code. However, without additional codes being registered, origins other than odontogenic (such as peritonsillar or submandibular origin) cannot be definitively excluded. When matching the patients in this study with the clinical data available to us from one of the hospitals involved (SU), 89% of the patients who were coded as K12.2 as a single diagnosis had DNSI of odontogenic origin. We excluded patients with DNSI codes related to submandibular gland and tonsils.

The mean number of collected prescriptions in relation to the DNSI hospitalisation was three. In some cases, patients with DNSI have received antibiotics before admission to the hospital and most of the patients were discharged with oral antibiotics after receiving intravenous antibiotics at the hospital. Oral antibiotics are relatively inexpensive, with a modest mean cost of 67 EUR (715 SEK) per patient. Only 40 of the 148 patients attended a follow-up visit to outpatient specialist ORL care. The likely reason for this is that patients with DNSI of odontogenic origin are seen either in specialised dental care or by an ORL surgeon at follow-up.

Prevention of DNSI is key to minimising patient suffering and significantly reducing resource utilisation and expenditure. Dental infections and dental procedures are common in all age groups, in both men and women. In this context, it is interesting that this study was conducted in a country with one of the world’s most generous public dental healthcare programmes, where all citizens have access to regular, reimbursed care from a public dentist. Two previous studies on COI for patients with DNSI were from countries with mainly private dental care programmes, and they showed similar results. For comparison, a dental procedure such as a difficult tooth extraction has a reference price of 156 EUR [1,655 SEK (2023)] in Sweden. If it develops into a DNSI the total COI corresponds to 99 difficult tooth extractions. Therefore, early treatment and prophylaxis are of fundamental importance for decreasing resource wastage.

Some of the data in this study had to be estimated. The data on oral surgery are not included in the CPP and data on short-term sick leave (< 14 days) were not available to us. The cost per hour of oral surgery is based on the average income of dental staff during office hours, even though treatments are often administered after office hours, at nights and during weekends. This could have led to an underestimation of the real costs. The majority of the patients in this study were of working age, and we calculated the in-hospital stay as a period of sick leave for the patients. However, we did not have any data on sick leave days beyond that, except for 12 patients. These 12 patients were registered for sick leave benefits at the Swedish Social Insurance Agency, extending the first 14 days; for them, the entire sick leave period could be calculated. It is unlikely that few, if any, of the other patients went straight back to work from the hospital. Accordingly, the indirect costs calculated in this study, representing 7% of the total COI, can be expected to be somewhat higher than 166,010 EUR (1,757,835 SEK) per patient.

## Limitations

Missing data and the risk of misreporting ICD codes are potential risks in a register-based study, since the registers and databases rely on information that is collated from administrative systems [14]. These errors can lead to inaccurate or inconsistent information being entered in the register and can have negative impacts on the data quality and reliability of the analyses and research based on these codes. Finally, healthcare costs paid directly by the patients are not included in this COI analysis, which may underestimate the economic burden of DNSI.

## Conclusion

The estimated COI for DNSI of odontogenic origin in this study was 2.28 million EUR (24,180,000 SEK), with a mean CPP of 15,400 EUR (163,400 SEK) per patient. Direct costs accounted for 93% of the expenses, primarily driven by inpatient care, while indirect costs made up 7% of the cost, being mainly related to production losses during sick leave. Deep neck space infections of odontogenic origin is a potentially preventable severe complication associated with dental diseases and procedures, and it results in high costs for healthcare systems.

## References

[1] Sheikh Z, Yu B, Heywood E, et al. The assessment and management of deep neck space infections in adults: a systematic review and qualitative evidence synthesis. Clin Otolaryngol. 2023;48(4):540–562. 10.1111/coa.14064. 37147934

[2] Rautaporras N, Uittamo J, Furuholm J, et al. Deep odontogenic infections – computed tomography imaging-based spreading routes and risk for airway obstruction. J Stomatol Oral Maxillofac Surg. 2023;124(4):101424. 10.1016/j.jormas.2023.101424. 36781108

[3] Patterson HC, Kelly JH, Strome M. Ludwig’s angina: an update. Laryngoscope. 1982;92(4):370–378. 10.1288/00005537-198204000-00003. 7070177

[4] Larawin V, Naipao J, Dubey SP. Head and neck space infections. Otolaryngol Head Neck Surg. 2006;135(6):889–893. 10.1016/j.otohns.2006.07.007. 17141079

[5] Lee JJ, Hahn LJ, Kao TP, et al. Post-tooth extraction sepsis without locoregional infection – a population-based study in Taiwan. Oral Dis. 2009;15(8):602–607. 10.1111/j.1601-0825.2009.01596.x. 19619196

[6] Kauffmann P, Cordesmeyer R, Troltzsch M, et al. Deep neck infections: a single-center analysis of 63 cases. Med Oral Patol Oral Cir Bucal. 2017;22(5): e536–e541. 10.4317/medoral.21799. 28809368 PMC5694174

[7] Eisler L, Wearda K, Romatoski K, et al. Morbidity and cost of odontogenic infections. Otolaryngol Head Neck Surg. 2013;149(1):84–88. 10.1177/0194599813485210. 23585157

[8] Marioni G, Rinaldi R, Staffieri C, et al. Deep neck infection with dental origin: analysis of 85 consecutive cases (2000–2006). Acta Otolaryngol. 2008;128(2):201–206. 10.1080/00016480701387157. 17851946

[9] Gralén K, Hjalte F, Persson U. Hälso- sjukvårdsutgifternas utveckling i Sverige. 2019. Contract No.: e-ISSN: 1651-8179.

[10] Biron VL, Kurien G, Dziegielewski P, et al. Surgical vs ultrasound-guided drainage of deep neck space abscesses: a randomized controlled trial: surgical vs ultrasound drainage. J Otolaryngol Head Neck Surg. 2013;42(1):18. 10.1186/1916-0216-42-18. 23672735 PMC3651187

[11] Han J, Liau I, Bayetto K, et al. The financial burden of acute odontogenic infections: the South Australian experience. Aust Dent J. 2020;65(1):39–45. 10.1111/adj.12726. 31618789

[12] SALAR. Nationella KKP-Principer. Ver 4. Kostnad Per Patient. ISBN: 978-91-7585-881-4. SKR; 2020.

[13] Olofsson S, Hjalte F. Produktionsbortfall – en metodologisk genomgång och beräkningar. Report No.: ISSN:1651-7598. Lund: IHE; 2020.

[14] Thygesen LC, Ersboll AK. When the entire population is the sample: strengths and limitations in register-based epidemiology. Eur J Epidemiol. 2014;29(8):551–558. 10.1007/s10654-013-9873-0. 24407880

